# Stroke in Ghana: A Situational Analysis

**DOI:** 10.1155/srat/1622597

**Published:** 2025-09-27

**Authors:** Samuel Darkwah, Aaron Awere-Duodu, Bismark Opoku-Asare, Eric S. Donkor

**Affiliations:** ^1^Department of Medical Microbiology, University of Ghana Medical School, Accra, Ghana; ^2^Department of Medicine and Therapeutics, Korle-Bu Teaching Hospital, Accra, Ghana

**Keywords:** comorbidities, complications, rehabilitation, stroke

## Abstract

Stroke ranks among the Top 3 leading causes of death and disability in Ghana. This review examines the current state of stroke in the country, focusing on recent developments and challenges in stroke care and rehabilitation. Historical and contemporary research indicates a rising prevalence of stroke-related morbidity and mortality, accompanied by a gradual shift from hemorrhagic to ischemic stroke, attributed mainly to the increasing adoption of Western lifestyles. Significant challenges to effective stroke care and rehabilitation exist, suggesting a potential worsening of the situation if these challenges are not addressed. With stroke morbidity and mortality expected to increase in the coming decades, there is an urgent need for substantial investments in stroke care, particularly in training healthcare professionals and providing adequate facilities and resources. Additionally, a comprehensive review of government health policies and stakeholder initiatives is necessary to enhance the quality of stroke care and mitigate the growing burden of stroke in Ghana.

## 1. Introduction

According to the World Health Organization (WHO) in 1970, “stroke is rapidly developing clinical signs of focal (or global) disturbance of cerebral function, with symptoms lasting 24 h or longer, or leading to death, with no apparent cause other than of vascular origin” [1]. Recently, the American Stroke Association proposed a new definition of stroke, which states that “stroke is an episode of acute neurological dysfunction presumed to be caused by ischemia or hemorrhage, persisting ≥ 24 h or until death” [1, 2]. Globally, stroke is the second leading cause of mortality, claiming 5.5 million lives annually [1, 3]. The burden of stroke does not rest entirely with its high mortality but also with its high morbidity, which results in about 50% of survivors being left chronically disabled [1, 4, 5].

Until recently, stroke was deemed a disease of Western countries. However, a shift in the burden of stroke from high-income countries to low- and middle-income countries (LMICs) has been reported, with two-thirds of global stroke mortality cases occurring in sub-Saharan Africa [6]. This development further worsens the existing challenges of the sub-Saharan African region, which is afflicted with poverty and communicable diseases such as HIV/AIDS [7]. Research findings have revealed that the region experiences an annual stroke incidence rate of 316 cases per 100,000 people, a prevalence rate of 315 per 100,000, and a mortality rate of 84% [8]. The disability-adjusted life years (DALYs) attributable to stroke in sub-Saharan Africa are approximately seven times greater than those reported in high-income countries [9]. These trends suggest that a significant increase in the burden of stroke in sub-Saharan Africa is likely over the coming decades, primarily due to the region's shift from infectious diseases to noncommunicable diseases (NCDs), including stroke [10].

Ghana, a country in sub-Saharan Africa, has experienced significant changes in its stroke burden [11], reflecting trends observed across the region [12]. The lingering economic impact of the recent COVID-19 pandemic has further strained the healthcare system, particularly in areas requiring substantial investments, such as specialized stroke care. This has created considerable challenges for poststroke care nationwide. This paper reviews the current situation of stroke in Ghana, emphasizing recent developments and the challenges influencing stroke outcomes in the country.

## 2. The Stroke Burden in Ghana and Some Causal Factors

In Ghana, stroke is one of the leading causes of death and is likely the foremost contributor to disability [13]. A 5-year study conducted on autopsy cases at Korle-Bu Teaching Hospital (KBTH) revealed that stroke accounted for 11% of autopsy findings, with hemorrhagic stroke being the more prevalent type (61%) [13]. The findings also indicated that males had a higher likelihood of dying from hemorrhagic stroke compared to females. Additionally, a 1-year retrospective examination of admitted patients who suffered strokes at the Komfo Anokye Teaching Hospital found that strokes represented 9.1% of total medical admissions for adults and accounted for 13.2% of all medical deaths among adults [14]. More recently, a comprehensive analysis of stroke admissions and mortality over 30 years at the same hospital was conducted, revealing a total of 12,233 stroke admissions with an equal distribution between genders [15]. During this period, the rate of stroke admissions rose significantly from 5.32 per 1000 admissions in 1983 to 13.85 per 1000 admissions by 2010, representing an increase of 260%. Concurrently, stroke mortality rates also climbed from 3.40 per 1000 deaths to 6.66 per 1000 deaths over three decades, with an average 28-day mortality rate of 41.1%. The economic impact of stroke in Ghana is substantial, as nearly 48% of stroke survivors fall within the working-age population. Thus, stroke also hugely burdens families when breadwinners are involved [16].

Well-known risk factors for stroke, such as hypertension, diabetes, excessive alcohol intake, obesity, and cigarette smoking, tend to be on the rise in Africa [17]. However, the precise extent to which these risk factors contribute to stroke predisposition remains to be fully elucidated. This gap is attributable to the notable heterogeneity in risk factor profiles, prevalence, incidence, types, subtypes, natural history, and stroke outcomes across different geographical locations and ethnicities. In Ghana, for example, males reportedly have significantly higher age-standardized stroke mortality rates than females compared to other countries in the subregion [18]. The leading risk factors for stroke show regional variations globally. However, findings from relevant studies conducted in Africa report that there is no significant association between stroke and well-known risk factors such as psychosocial factors, diabetes, and physical inactivity [19, 20]. The identification and characterization of dominant risk factors for stroke and its subtypes in sub-Saharan Africa, particularly Ghana, are imperative to developing tailored, strategic interventions to mitigate the burden of stroke in Ghana and the African subregion. In line with efforts to assess stroke risk factors in Ghana, the Stroke Investigative Research and Education Network (SIREN) project, the largest stroke study in Africa, has identified the Top 11 potentially modifiable risk factors accounting for about 98% of population-attributable risk of stroke among Ghanaians [21]. The SIREN study report highlights dietary, lifestyle, and psychosocial factors as majorly associated with stroke in Ghana and underscores the significant effect of hypertension on stroke occurrence in the African population.

Several lines of evidence indicate that hypertension is the most important risk factor for stroke in Ghana. A cross-sectional study by Donkor et al. [22, 23] among 156 stroke survivors in Ghana reported the distribution of stroke risk factors as hypertension (89%), diabetes (29%), alcohol consumption (28%), high cholesterol (25%), obesity (24%), family history of cardiovascular disease (13%), atrial fibrillation (11%), and heart disease (7%). In a prospective cohort study by Sarfo et al. [24], a history of cigarette smoking (HR [95% CI]: 2.59 [1.18; 5.67]) and physical inactivity (1.81 [1.06; 3.10]) were found to be independently associated with stroke. The findings presented above indicate a trend of high and increasing stroke burden in Ghana. Hypertension is highly prevalent among the general population in Ghana, which could be contributing to the rising incidence of strokes in the country [15, 25, 26]. If left unaddressed, the public health burden of stroke in Ghana is expected to grow, underscoring the need for immediate action to manage hypertension effectively. Additionally, there is a significant number of individuals with undiagnosed hypertension, along with a low adherence rate to antihypertensive medications [27, 28]. These issues are critical, especially considering the established link between hypertension and stroke. Juxtaposing these findings with evidence from the SIREN study, it becomes evident that although hypertension seems to be the most important risk factor in Ghana and consequently the prime focus of stroke control strategies, other modifiable factors such as lifestyle and psychosocial factors require equal attention in strategic interventions for stroke control.

The significant prevalence of stroke in Ghana is, in part, attributable to a lack of community awareness regarding the risk factors and warning signs associated with the condition. Research indicates that heightened awareness of stroke risk factors can enhance adherence to preventative measures, while failure to recognize warning signs contributes to delays in seeking hospital care for strokes [23, 29, 30]. A study conducted in Accra, the capital of Ghana, found that most participants were unable to identify hypertension or other primary stroke risk factors, including high cholesterol, diabetes, alcohol consumption, and smoking [23, 30]. Similarly, many were unaware of fundamental warning signs of stroke, such as slurred speech and visual disturbances.

## 3. Stroke Subtypes and Their Evolution in Ghana

Strokes are divided into two main types: ischemic and hemorrhagic strokes. Ischemic stroke occurs when the blood supply to a specific area of the brain is interrupted, resulting in a sudden loss of function. Conversely, hemorrhagic stroke results from the rupture of a blood vessel or an abnormal blood vessel [1]. Globally, ischemic strokes represent approximately 80% of all stroke cases, while hemorrhagic strokes make up around 20% [1, 31]. Recent epidemiological data reveal that in Africa, ischemic strokes account for 66% of cases, with hemorrhagic strokes comprising 34%. This is in stark contrast to high-income countries, where ischemic strokes represent 91% and hemorrhagic strokes only 9% [32].

In Ghana, there has been a shift in the prevalence of stroke types, with a notable decrease in hemorrhagic strokes and a rising trend in ischemic strokes [13, 33]. Historical studies from 1954 and 1981 indicated that hemorrhagic strokes were the leading type, responsible for about 90% of stroke-related deaths in the country [1, 22]. However, retrospective data covering the years 1994–1998 indicated that the prevalence of hemorrhagic strokes had dropped to 61%, while ischemic strokes were becoming more prevalent [13]. The increased prevalence of ischemic strokes in recent years in African countries like Ghana may be linked to the adoption of Western lifestyles [34, 35]. Urbanization, globalization, and media influence are major contributors to Western lifestyle adoption in Ghana [36, 37] and other African countries such as Nigeria [38, 39], which have reported an increased prevalence of ischemic stroke in recent years [40]. Furthermore, there is a tendency to favor ischemic strokes in clinical discussions, as patients with ischemic strokes generally have better survival rates compared to those with hemorrhagic strokes.

## 4. Stroke Complications and Comorbidities

Comorbidity and complications are common among stroke survivors in Ghana and are associated with both increased incidence, worsened outcomes, and risk of mortality [17]. Stroke survivors, particularly the elderly, are commonly predisposed to multiple medical conditions and thus become multimorbid [41]. Atherosclerosis, hypertension, diabetes, and heart diseases are common preexisting or acquired conditions that impact patients and also share pathological mechanisms with stroke [42]. These conditions usually form the risk factors for cardiovascular and cerebrovascular events that can promote stroke recurrence. The presence of comorbidity in stroke patients negatively impacts their rehabilitation process, increases healthcare costs, and contributes to poorer functional outcomes overall [42, 43]. As mentioned earlier, generally, metabolic and cardiovascular risk factors such as hyperglycemia and hypertension are common comorbidities of stroke; however, a multitude of disorders that form a part of comorbid indices are associated with stroke. Such disorders include orthopedic disorders (e.g., fractures and degenerative disorders), psychiatric disorders, and vertigo [44].

Patients with acute stroke are at risk of developing a plethora of complications that can be life-threatening. A major poststroke complication is infections. Infections are prevalent in approximately 9.14% of stroke patients, a significant drop from 30%, with the most common poststroke infections being pneumonia and urinary tract infections (UTIs) [4, 45]. However, knowledge regarding these infections in stroke patients in Africa remains limited. Several clinical factors, such as baseline characteristics of patients (e.g., age and dysphagia), facilitate the development of poststroke infections. Importantly, the use of invasive procedures and devices like catheters and mechanical ventilators in the management of stroke patients significantly increases the risk, as well as the incidence of infections [46]. A study conducted at the KBTH in Ghana examined the epidemiology of bacteriuria among stroke patients, focusing on its prevalence, incidence, risk factors, and causative agents [47]. This longitudinal study included 55 outpatients and 16 inpatients with strokes, recruited from the physiotherapy clinic and the stroke admission ward at KBTH. Urine cultures were performed daily for inpatients until their discharge, while outpatients provided urine samples for analysis weekly or biweekly over 6 months. Demographic and clinical data were collected from the participants' medical records. The findings indicated that bacteriuria prevalence was 10.9% (six out of 55) among outpatients and 18.8% (three out of 16) among inpatients, with no statistically significant difference (*p* = 0.411). Throughout the follow-up period, there was one new case of bacteriuria in both groups. Overall, only one out of nine (11%) of the bacteriuria cases in stroke patients was symptomatic. Predictors for bacteriuria included severe stroke (odds ratio [OR] = 17.7, *p* = 0.008) and pyuria (OR = 38.7, *p* = 0.002). *Escherichia coli* was identified as the most prevalent organism responsible for bacteriuria, demonstrating susceptibility to amikacin but resistance to ampicillin, cefuroxime, tetracycline, cotrimoxazole, norfloxacin, meropenem, and augmentin. Bacteriuria was found to be a common complication among both inpatient and outpatient stroke patients at KBTH, with a higher prevalence observed in inpatients. Stroke severity emerged as the primary stroke-related factor associated with bacteriuria, which was predominantly asymptomatic, with *E. coli* being the key causative agent.

Similarly, another study by Donkor et al. [48] assessed the risk of community-acquired bacteriuria among stroke patients, alongside any related factors and the etiologic organisms. This study involved 70 stroke patients and 83 healthy controls, matched by age and sex, in a cross-sectional design. Urine samples were obtained from all participants and analyzed using standard microbiological techniques. Demographic and clinical data were collected, including specific details on stroke duration, frequency, and subtype. The results revealed a significantly higher prevalence of bacteriuria in stroke patients (24.3%) compared to controls (7.2%), with a relative risk of 3.36 (1.40–8.01) (*p* = 0.006). All six bacteriuria cases in the control group were asymptomatic, whereas among stroke patients, 15 were asymptomatic and two were symptomatic. Significant associations were identified between bacteriuria and female sex (OR: 3.40 [1.12–10.30], *p* = 0.03) as well as the presence of stroke (OR: 0.24 [0.08–0.70], *p* = 0.009). The bacterial profiles in both groups were similar, with coagulase-negative *Staphylococcus* species being the most frequently isolated organism, occurring in 12.9% of stroke patients and 2.4% of controls. Stroke patients in these studies exhibited a significantly increased risk of community-acquired bacteriuria, which was generally asymptomatic and had little or no correlation with clinical parameters such as stroke duration, frequency, and subtype [47, 48]. Interestingly, these findings reveal that there is no relationship between poststroke bacteriuria and the clinical characteristics of stroke, such as stroke duration, frequency, and subtype.

Immunological impairments are other factors that increase the risk of infection complications among stroke patients. Immune suppression or dysfunction might stem from other stroke comorbidities, such as diabetes or stroke-induced impairments. There is a correlation between some inflammatory markers and the development of poststroke infections. Low levels of TNF-*α* and high levels of IL-10, IL-6, and IL-1*β* have been shown to correlate with the development of poststroke infections [46, 49, 50]. Poststroke immunosuppression is strongly associated with susceptibility to infections among stroke patients. The pathophysiological events of stroke decrease lymphocyte counts in circulation by altering lymphoid lineage progression in the bone marrow of stroke patients [51]. Stroke induces loss of function in monocytes and thus renders them incapable of providing adequate costimulation required for T lymphocyte activation [52]. In Ghana, no immunomodulatory therapy has been clinically tested and approved for managing poststroke infection, and data on immunotherapy for stroke management in the region are scarce. The effective regulation of stroke-induced immune suppression and dysfunction could be of clinical and therapeutic relevance for the prevention of poststroke complications. Immunoregulatory approaches aimed at mitigating early proinflammatory dynamics in stroke might be promising therapies to limit the complications of infections after stroke.

Diabetes mellitus (DM) is a well-known independent risk factor and a common complication of stroke. About 25% of stroke survivors have been diagnosed with DM as a comorbidity, comparable with global estimates of about 28% [41, 53]. However, this prevalence drastically rises to about 60% when undiagnosed DM is considered [41]. This implies that there is a high burden of stroke survivors with undiagnosed prediabetes and/or DM. This burden significantly heightens the risk for severe cardiovascular disease and associated complications. The association between stroke complicated by DM and infections remains debatable, and research reporting this association is limited. Evidence suggests that DM is significantly associated with an increased risk of poststroke urinary infections and pneumonia [54]. Patients with diabetes may exhibit glycosuria, which can promote the growth of uropathogens responsible for urinary infections. Additionally, DM may elevate the risk of UTIs and other infections due to compromised immune function caused by hyperglycemia and diabetes-related microangiopathy [55].

Hypertension, with its associated disorders, is a comorbidity of stroke and is a very important modifiable risk factor that drives cerebral small vessel disease and negatively impacts cerebral circulation and cognitive function [56, 57]. In Ghana, hypertension has been identified as the main risk factor for stroke, and this could be attributed to the rapid economic development and urbanization [47, 48]. A rare phenotype of resistant hypertension known as refractory hypertension is found among approximately 2.2%–5.8% of stroke survivors in Ghana, with an increased prevalence observed among survivors of the hemorrhagic stroke subtype [58]. Thus, refractory hypertension is more frequently observed among stroke survivors in Ghana and increases their risk of mortality. Additionally, there is an eightfold higher risk of refractory hypertension compared with stroke-free individuals with hypertension [58]. It is estimated that about 5%–15% of stroke patients will develop seizures within 2 years of stroke onset [59]. Thus, stroke largely contributes to symptomatic seizures among older adults, and the risk factors identified for this include the use of antihypertensive treatments, stroke subtype and location, and poststroke persistent disabilities [59–61].

## 5. Stroke Care and Rehabilitation in Ghana

Generally, tertiary hospitals in Ghana are better equipped with modern stroke care services than regional hospitals, although such services may be less accessible in the northern regions of the country [29, 62, 63]. There has been a rise in herbal centers offering alternative medical treatments for various conditions, including stroke [29, 64]. Ghana pioneers a groundbreaking milestone in West Africa with the establishment of a dedicated stroke unit in the largest teaching hospital in its capital, Accra. Presently, there is a national initiative in collaboration with Wessex Hospital in the United Kingdom aimed at enhancing stroke care in Ghana [65]. This collaboration between professional health workers from Ghana and the United Kingdom initially prioritized the development and establishment of a multidisciplinary stroke unit at Greater Accra's largest tertiary healthcare facility, KBTH. The Ho Teaching Hospital in the eastern belt inaugurated a specialized stroke and private ward in March 2025, and the Tamale Teaching Hospital in the northern belt has the development of a stroke unit underway. The establishment of organized and dedicated stroke units merits a favorable outcome and reduces the burden of stroke [66, 67]. However, it is not known whether the establishment of the stroke unit in Ghana has reduced the rates of stroke. Although few studies have hinted at a high burden of stroke in Ghana [25], assessing the impact of the stroke unit on stroke rates can be challenging since the general burden of stroke in Ghana is not known; there is a lack of extensive research and reports on stroke in Ghana. Nonetheless, studies have shown that stroke patients treated in specialized units for stroke management do better than those treated in general medical wards. Thus, stroke units increase survival rates among stroke patients [68]. Hospitals with stroke units have shorter response times from patient arrival to diagnostic imaging following the onset of stroke symptoms [69]. It is worth mentioning that the KBTH stroke unit seems to have faced several hurdles in terms of political and institutional support since its development. Adequate care for stroke patients in Ghana leaves more to be desired in the areas of the availability of resources; the lack of beds in care facilities, coupled with the scarcity of therapists, allows for only the severest stroke cases to be admitted and cared for [70]. A systematic review by Kankam et al. [11] reported that stroke rehabilitation in Ghana faced notable challenges, including poor knowledge of stroke rehabilitation among health professionals and variations in the guidelines and resources for stroke management at the different levels of health facilities. Similar findings were also reported by Baatiema et al. [29], where factors such as inadequate health facilities, lack of stroke care protocol, limited staff numbers, insufficient staff development avenues, poor collaboration between health professionals, limited knowledge of stroke care interventions among health professionals, and lack of political will to implement broader national health policy levels.

Stroke diagnosis in Ghana faces several challenges. Diagnostic and assessment services such as CT scans and MRI are accessible at regional and tertiary healthcare facilities; however, they have limited functional and operational availability. Brain CT scanning is available for 24-h, all-week-long service in only 18.2% of hospitals, and MRI scan services, including other advanced neurovascular diagnostic services, are functional in only 27% of hospitals [29]. The trends in stroke admissions and mortality are high in Ghana, with a 28-day mortality rate as high as 41% reported in the central part of Ghana [15]. The burden of stroke also keeps increasing, with a 1-month in-hospital case fatality as high as 41%–43%. These statistics warrant the need to improve the quality of care for stroke. Data on the quality of care for stroke in Ghana is limited; however, a few surveys have described the quality of care for stroke patients in Ghana and have identified significant gaps in the assessment, diagnosis, acute management, secondary prevention, and rehabilitation of stroke [29, 71]. The most probable reason for this is the lack of functional stroke units and neurologists. Further studies are needed to assess the quality of care for stroke management in Ghana.

Ghana's National Health Insurance Scheme (NHIS) was established in 2003 to provide equitable access to healthcare to its citizens [72]. It covers outpatient services and some inpatient services, as well as diagnostic services and essential medications [73]. However, healthcare services for some NCDs like stroke are not covered by the scheme. This aggravates the financial burden on stroke patients and their families. On a positive note, the government of Ghana in 2025 launched the Ghana Medical Trust Fund, a health fund created to cover the cost of care and medications for NCDs such as hypertension, stroke, and cancer, which were not covered under the NHIS introduced in 2003 [74]. However, its implementation is yet to be done. While Ghana's per capita health expenditure of $75 is lower than the African average of $128 per capita, the worsening economic situation over the years has led to high healthcare costs, particularly for individuals living with chronic conditions [75]. A study by Atibila et al. reported that the total patient cost for stroke was GH₵ 4647.40, with the weighted average medication and treatment cost being GH₵ 4464.00 [75]. Additionally, the study estimated that the lifetime cost of four complications of hypertension, including stroke, was GH₵ 869,106.00 (GH₵ 570,239.00–GH₵ 1.202 million). These estimations are suggestive of the high cost of stroke care in Ghana and highlight the financial burden on stroke patients and their families.

Pluralistic healthcare is common among stroke survivors and their caregivers in Ghana. This practice, coupled with other factors such as poor healthcare accessibility and financial constraints, may complicate the effective management of stroke cases [29, 76–78]. Collaborative efforts to discuss stroke care in Ghana by researchers, healthcare providers, caregivers, and stroke patients from institutions in the United Kingdom and Ghana have yielded practical recommendations for improvement [79]. These proposed stroke care improvement strategies emphasize the need for the government and relevant stakeholders to build research capacity in herbal medicines for stroke treatment and the strengthening of the Centre for Plant Medicine Research of the Council for Scientific and Industrial Research (CSIR) in Ghana to spearhead this effort. Additionally, there is a need to increase the number of trained health professionals in stroke care and rehabilitation services to bridge the gap in the inadequate stroke workforce in the country. The coverage of national health insurance policies may also need to be expanded to cover stroke management and rehabilitation. By doing so, challenges in stroke care, such as delayed diagnosis and treatment of stroke and nonadherence to medication and rehabilitation therapies, could be mitigated [80].

Ghana's commitment and preparedness to improve healthcare and reduce the burden of acute stroke leave more to be desired. There is a significant deficit in national support for acute stroke care, as well as limited funding from hospital management to support stroke care [81]. Not many hospital or community stroke awareness programs and state-supported community stroke rehabilitative programs are available. Consequently, poststroke care and long-term rehabilitative care following inpatient care at the hospital often become the sole responsibility of families and caregivers [29]. Having highlighted the need to improve healthcare and reduce the burden of stroke in Ghana, it is worth mentioning that the Ghanaian government has demonstrated some efforts. In August 2012, the Ministry of Health published Ghana's maiden National Policy for the Prevention and Control of Chronic NCDs (NCD policy) with the mission to prevent and control avoidable NCD-related morbidity and mortality through various local and international framework policy strategies [82]. The policy and strategy addressed key aspects of the stroke burden in Ghana, particularly the major risk factors of stroke as a NCD. This maiden policy achieved several successes during its period of implementation. For example, the NCD policy ensured the passing of legislative instruments on tobacco control regulations, the development of a national alcohol policy, the inclusion of diabetes and hypertension in Ghana's integrated disease surveillance and response (IDSR), and the inclusion of some NCDs in the benefits package of the national health insurance policy. Although stroke and cerebrovascular accidents (CVAs) are not captured as “special concern” NCDs in Ghana's maiden and current NCD policy document, the objectives and strategies cover key risk factors for stroke. Thus, the current policy seeks to prevent NCDs (including stroke) by reducing exposure to risks (physical inactivity, tobacco use, alcohol use, poor dieting, and nutrition) through public education, risk factor surveillance, vaccination and immunization programs, and provision of a safe, enabling environment for physical activities [83]. The NCD policy has been revised twice, in 2016 and 2022, respectively [84]. The most recent update in 2022 saw the establishment of a broader long-term policy, which emphasizes multisectoral collaboration between government, the private sector, civil society organizations, and development partners to manage the NCD burden. To ensure its sustainability, the revised policy has been aligned with international policies such as the universal health coverage and Sustainable Development Goal 3.4, which seeks to reduce premature NCD deaths by a third by 2030. Although Ghana's NCD policy somewhat addresses stroke indirectly, the lack of a stroke-specific health policy may be a setback in efforts to address the stroke burden. This indicates that Ghana has yet to manifest its commitment to reducing the global stroke burden in its health policy.

Efforts to improve stroke care and management in Ghana have not been the sole mandate of the government. The Ghana Heart Foundation, for example, is a nongovernmental organization that is pivotal in providing health education on stroke and other cardiovascular diseases [30]. Some private establishments, in recent times, also offer stroke care in specialized private stroke units and rehabilitation centers. One such facility is the Euracare Advanced Diagnostic Health Center, which has an acute stroke unit facility in Accra that offers specialized stroke care. Additionally, some private hospitals staffed by neurologists and specialists contribute to providing specialized stroke care, mostly in urban areas of the capital. Despite the efforts of private facilities to reduce stroke rates in Ghana, the high cost of stroke management in a private facility, coupled with limited accessibility to these facilities in rural areas, remains a significant challenge. Other challenges include limits on national health insurance coverage in private facilities and disparities in the quality of care across different private hospitals.

Considering that many stroke survivors fall within the working age group, it is important to ensure their reintegration into the communities to function productively [16, 85]. This underlies the main aim of rehabilitation of stroke survivors. In Africa, medical aid and physiotherapy services make up the main rehabilitation services available to stroke patients, and this is also the case in Ghana, where several stroke survivors engage in some form of physiotherapy service in addition to medical check-ups [86, 87]. Ghana's stroke care and rehabilitation prominently include a pluralistic system comprising both conventional (biomedical personnel) and complementary (e.g., herbal and faith healers) health providers. Most patients with stroke resort to complementary medicine treatment as an exclusive management option or in conjunction with conventional medicine [88]. However, there is limited data on stroke rehabilitation and its impact on the social and community life of stroke survivors in Ghana. Several months of rehabilitation tend to improve stroke survivors' ability to self-care and be somewhat independent in their daily living [86, 89]. Kankam et al. [11] in their systematic review on stroke rehabilitation in Ghana reported significant benefits from telerehabilitation, with improved physical and cognitive outcomes attributed to increased participation in rehabilitation activities among stroke survivors. However, many rehabilitated stroke survivors in Ghana are unable to return to work due to residual mobility impairments. For instance, studies by Opoku et al. [89] and Mohammed et al. [86] revealed that stroke survivors were unable to return to work or enjoy social activities despite reporting improved functionality from rehabilitation. Similar findings were reported in South Africa [90] and Malawi [91], highlighting the similarities in challenges faced by stroke survivors in Africa. This precipitates the need to incorporate condition-friendly vocational and occupational facets into rehabilitation programs for stroke survivors in Ghana and the need to provide supportive social and working environments available in high-income countries, such as Sweden [92] and the Netherlands [93]. A randomized control trial in South Africa, which assessed the effect of a workplace intervention program on return to work after stroke, reported a 60% return rate among the intervention group compared to a 20% rate in the control group, substantiating the findings of such interventions in high-income countries [94]. The effectiveness of occupational therapy in stroke rehabilitation in Ghana remains a gray area and warrants exploration by future studies. Several challenges, such as financial constraints, influence stroke patients and their caregivers' decisions to use conventional and complementary rehabilitation as the main treatment for stroke. This choice is usually based on the availability of facilities in the communities and the acceptability of their health or religious beliefs [78, 95].

## 6. The Stroke Workforce in Ghana

### 6.1. Multidisciplinary Team Working

Stroke care requires good collaboration between multidisciplinary healthcare teams comprised of physicians, nurses, radiologists, physiotherapists, psychologists, and dietitians, among other health professionals; this is essential in achieving positive treatment outcomes [96]. At the KBTH in Accra, a multidisciplinary team training organized by health professionals from Wessex through the Wessex-Ghana Stroke Partnership led to a reduction in aspiration pneumonia rates [65, 97]. A study by Mohammed et al. evaluated the composition of stroke rehabilitation teams at a primary, a secondary, and a tertiary hospital in the Greater Accra Region [98]. At the KBTH (tertiary), the team consisted of physiotherapists (16%), clinical psychologists (3%), nurses (52%), occupational therapists (3%), dieticians (2%), and doctors (24%). At the secondary hospital (Tema General Hospital), the team was made up of physiotherapists (9%), nurses (29%), dietitians (8%), and doctors (54%). Lastly, the Amasaman District Hospital, a primary facility, had doctors (7%), physiotherapists (14%), and nurses (79%) in their team. Despite the varying compositions of the various stroke teams, the structure and process of stroke rehabilitation were similar across the hospitals. However, poor coordination in even well-constituted teams can affect the quality of care rendered. There have also been reports of personnel shortages in vital roles, such as speech and language therapy, in most hospitals in Ghana [11, 98]. These limitations to stroke rehabilitation care in Ghana might contribute to the limited uptake of stroke care services and facilities [81], consistent with some reports on stroke services in Africa [99].

### 6.2. The Role of the Brain Drain and Current Stroke Workforce Changes

The migration of medical specialists and physicians could significantly impact the strength and availability of human resources in managing stroke in Ghana. A systematic review of the emigration of physicians from West Africa identified reasons for the increasing emigration of physicians [100]. The main reasons for emigration included poor working conditions, such as limited infrastructure, staff, and equipment; limited career opportunities; low standards of living; and political unrest. A case study by Owusu-Ansah conducted in three major hospitals in Accra, namely, the KBTH, the 37 Military Hospital, and the Ridge Hospital, investigated why Ghanaian doctors and nurses emigrate [101]. The results indicated that the overall satisfaction with working conditions, incentive packages, family relations, and societal recognition as health professionals significantly influenced interest in emigration by the participants of both professions. Another study exploring the extent of the brain drain of Ghanaian radiographers, who play an essential role in stroke diagnosis, revealed that poor salary and poor working conditions facilitated their exodus [102], which is in agreement with those of physicians and nurses reported by other studies [101, 103]. Additionally, the recent COVID-19 pandemic exposed the depth of emigration by Ghanaian physicians and nurses when the existing workload increased, causing a reduction in available services for specialized care, including stroke care [104].

## 7. Conclusion

Stroke in Ghana still ranks high among the top causes of mortality and morbidity, and efforts to reduce its social and economic burden, as well as the quality of care for survivors, are desired. Trends in the evolution of stroke subtypes in Ghana reveal a significant decline in hemorrhagic stroke and an increase in ischemic stroke, respectively. Most stroke patients are multimorbid, and complications such as hypertension, diabetes, heart diseases, and orthopedic complications are common among stroke survivors. There is a need for a critical look at stroke care and rehabilitation and to assess the impact of government health policies, as well as various efforts by stakeholders to improve the quality of care for stroke in Ghana. While the 2022 NCD policy emphasized health promotion and education on NCD risk factors, the issue of health worker migration was not addressed. Owing to the increase in health worker migration in recent years, broader discussions and implementation of measures are needed to curb this issue and ensure the availability of an adequate health workforce for specialized care services such as stroke care. Moreover, the establishment of stroke units by the government in district, municipal, and regional hospitals is required to increase the availability and accessibility of specialized stroke care in the country. Additionally, regular training of multidisciplinary stroke teams is required to enhance the delivery of high-quality stroke care and rehabilitation services aimed at achieving desired treatment outcomes.

## Data Availability

Data sharing is not applicable to this article as no datasets were generated or analyzed during the current study
